# Evaluation of renal damage in a bleomycin-induced murine model of systemic sclerosis

**DOI:** 10.22038/IJBMS.2023.67117.14720

**Published:** 2023

**Authors:** Dulce Carolina Pérez-Figueroa, Edilburga Reyes-Jiménez, Juan Manuel Velázquez-Enríquez, Itayetzi Reyes-Avendaño, Karina González-García, Saúl Villa-Treviño, Honorio Torres-Aguilar, Rafael Baltiérrez-Hoyos, Verónica Rocío Vásquez-Garzón

**Affiliations:** 1 Laboratorio de Fibrosis y Cáncer, Facultad de Medicina y Cirugía, Universidad Autónoma Benito Juárez de Oaxaca, Oaxaca, México; 2 Departamento de Biología Celular, Centro de Investigación y de Estudios Avanzados del Instituto Politécnico Nacional, Ciudad de México, México; 3 Facultad de Ciencias Químicas, Universidad Autónoma Benito Juárez de Oaxaca, Oaxaca, México; 4 CONACYT, Facultad de Medicina y Cirugía, Universidad Autónoma Benito Juárez de Oaxaca, Oaxaca, México

**Keywords:** Bleomycin, Fibrosis, Kidney, Oxidative stress, Scleroderma, Scleroderma renal crisis, Systemic sclerosis

## Abstract

**Objective(s):**

Systemic sclerosis (SSc) is an autoimmune disease of unknown etiology with a high mortality rate. Renal crisis has been reported as one of the predictors of early mortality in these patients. The present study was performed to evaluate bleomycin-induced SSc using an osmotic minipump as a possible model for the analysis of renal damage in SSc.

**Materials and Methods:**

Male CD1 mice were implanted with osmotic minipumps loaded with saline or bleomycin and sacrificed at 6 and 14 days. Histopathological analysis was performed through hematoxylin and eosin (H&E) and Masson’s trichrome staining. The expression of endothelin 1 (ET-1), inducible nitric oxide synthase (iNOS), transforming growth factor β (TGF-β), and 8-hydroxy-2-deoxyguanosine (8-OHdG) was also evaluated by immunohistochemistry.

**Results:**

The administration of bleomycin induced a decrease in the length of Bowman’s space (3.6 μm, *P*<0.001); an increase in collagen deposition (14.6%, *P*<0.0001); and an increase in the expression of ET-1 (7.5%, *P*<0.0001), iNOS (10.8%, *P*<0.0001), 8-OHdG (161 nuclei, *P*<0.0001), and TGF-β (2.4% µm, *P*<0.0001) on Day 6. On Day 14, a decrease in the length of Bowman’s space (2.6 μm, *P*<0.0001); increased collagen deposition (13.4%, *P*<0.0001); and increased expression of ET-1 (2.7%, *P*<0.001), iNOS (10.1%, *P*<0.0001), 8-OHdG (133 nuclei, *P*<0.001), and TGF-β (0.6%, *P*<0.0001) were also observed.

**Conclusion:**

Systemic administration of bleomycin via an osmotic minipump produces histopathological changes in the kidneys, similar to kidney damage in SSc. Therefore, this model would allow the study of molecular alterations associated with SSc-related renal damage.

## Introduction

Systemic sclerosis (SSc) or scleroderma is an autoimmune disease of unknown etiology with a distinctive pathogenic hallmark characterized by immune dysregulation, microvascular damage, and widespread fibrosis in multiple organs (1). SSc is characterized by the development of cutaneous fibrosis; however, it can affect other organs, such as the lungs, heart, gastrointestinal tract, liver, and kidneys (2). Although SSc is classified as a rare disease, it has a high morbidity and mortality rate because approximately 50% of diagnosed patients die due to multiorgan complications (3, 4). In addition, the high mortality rate of SSc is mainly associated with the development of interstitial lung disease, which is one of the most critical complications (5). However, autopsy studies in patients with SSc showed occult renal damage in approximately 80% of patients, and it has been hypothesized that renal damage remains subclinical until the advanced stages of the disease (6). Additionally, renal damage in patients with SSc manifests in chronic forms of renal failure and scleroderma renal crisis (SRC) (7). Furthermore, a multinational cohort study of SSc subjects reported that renal crisis is one of the predictors of early mortality in these patients (8). However, despite increasing efforts to characterize the disease, the pathogenesis of SSc and renal involvement remain poorly understood.

Vasculopathy and subsequent immune activation leading to fibroblast activation and fibrosis are suggested as the final effects of these processes (9). Additionally, mediators of vascular tone, including endothelins and nitric oxide (NO), have been described to play a crucial role (10-12). On the other hand, TGF-β is one of the critical cytokines for fibrosis development, which stimulates fibroblasts to differentiate into myofibroblasts, producing large amounts of extracellular matrix (ECM) (13).

Animal models are currently a fundamental tool to study different chronic diseases, such as SSc, to better describe the pathogenesis of the disease (14). In this regard, the bleomycin-induced SSc model is the most widely used to study the condition due to its great capacity to promote fibrosis development (15, 16). Bleomycin (BLM) is part of the glycopeptide antibiotic family and exerts an antitumor effect against various human tumors. Unfortunately, BLM induces adverse side effects, such as the development of pulmonary fibrosis (17). BLM causes damage by binding directly to DNA or by increasing the production of reactive species. Greater toxicity has been observed in tissues such as the lungs and skin due to the low activity of bleomycin hydrolase, the enzyme responsible for the inactivation of BLM (17, 18). In addition, other organ involvement has been observed, including the liver, and this has been related to the systemic effects generated by increased oxidative stress and inflammation (16, 19).

Therefore, this drug has been widely used in experimental models to induce fibrosis in various tissues. Of note, the dose and route of BLM administration lead to different effects, with the advantage of reproducing fibrosis models in the skin, lung, and liver (16, 19). When BLM is administered continuously using an osmotic minipump, it can mimic the skin and lung fibrosis suffered by patients with SSc (20). Therefore, it is possible to speculate that other organs, such as the kidney, may be damaged by BLM administration.

The present study was conducted to evaluate BLM-induced SSc using an osmotic minipump as a possible model for the analysis of renal damage in SSc, focusing in particular on histological alterations; collagen deposition; and the expression of proteins involved in microvasculopathy, oxidative stress, inflammation, and fibrosis.

## Materials and Methods

***BLM-induced SSc model***

Male CD1 mice were acquired from the Laboratory Animal Production and Experimentation Unit of the Center for Research and Advanced Studies of the National Polytechnic Institute (UPEAL-CINVESTAV-IPN). All experiments were carried out in accordance with the ethical principles of animal experimentation under the approval of the Institutional Animal Care and Use Committee (IACUC) of CINVESTAV-IPN (protocol 0109-14). During the experimental procedures, the mice were fed *ad libitum *and maintained under temperature conditions of 22 ± 3 °C in a controlled environment with 12 hr light/dark cycles. The BLM-induced SSc model was used. Mice were randomly divided into four experimental groups (4 mice per group). For each day of sacrifice (6 and 14 days), a control group (CT) was administered saline solution, and a bleomycin group (BLM) was administered the BLM drug as previously reported (19). Briefly, an osmotic minipump (ALZET 1007D, DURECT, Cupertino, CA, USA) containing saline as a vehicle or 100 U/kg BLM (Teva Parenteral Medicines, Irvine, CA, USA) was used. The osmotic minipump was designed to deliver its contents at a rate of 0.5 μl/hr for seven days and was implanted subcutaneously in the scapular area under isoflurane anesthesia.

The pumps were removed at day ten as recommended by the manufacturer, and the mice were sacrificed under deep anesthesia at 6 and 14 days of treatment after implantation. Then, the skin, lungs, and kidneys were collected for subsequent analyses.

***Tissue processing and histological analysis***

Tissue samples were processed for histological and immunohistochemical analysis. After sacrifice, the skin, lungs and kidneys were removed and dehydrated in a series of alcohol and xylol concentrations for embedding into paraffin. Tissue sections of 5 μm for histological analyses and 3 μm for immunohistochemical analyses were sectioned using a microtome (Leica, model RM 2125 RTS). Tissue sections for histological analysis were recovered on gelatinized slides and for immunohistochemical analysis on silanized slides.

Tissue sections were stained with hematoxylin and eosin (H&E) to observe histological alterations. Briefly, after deparaffinization at 56 °C, the slides were rehydrated in decreasing concentrations of xylol and alcohol and finally tap water; immersed in Harris’ hematoxylin solution (738, HYCEL, Jalisco, Mexico), acid alcohol, ammonia solution, and yellowish eosin (688, HYCEL, Jalisco, Mexico); and finally dehydrated and mounted with synthetic resin.

Masson’s trichrome staining (HT15 kit, Sigma‒Aldrich, St. Louis, MO, USA) was performed to evaluate collagen deposition. For this purpose, after deparaffinization and hydration, the slides were immersed in Bouin’s solution and subsequently washed. Then, they were stained with Weigert’s ferric hematoxylin, washed, stained with Biebrich’s acid fuchsin scarlet and washed again. Subsequently, the slides were treated with a solution of phosphotungstic acid and phosphomolybdic acid, stained with aniline blue, treated with acetic acid, dehydrated and mounted in synthetic resin. After mounting, all tissues were observed at 10x and 40x magnification under an optical microscope (Primo Star, CARL ZEISS). Image analysis for the quantification of the percentage of positive areas for collagen was performed with the ImageJ v.2.3.0/1.53f software (U. S. National Institutes of Health, Bethesda, Maryland, USA), using the Masson Trichrome vector as previously described (21).

***Immunohistochemical analysis***

The 3 µm sections were recovered on silanized slides, deparaffinized at 56 °C, and then rehydrated. Antigen retrieval was performed in citrate buffer (pH 6.0). Sections were incubated in a humidified chamber with 3% BSA to block nonspecific binding. The sections were then incubated at 4 °C overnight with the corresponding primary antibodies diluted in 1% BSA as follows: rabbit polyclonal anti-TGF-β (1:100; sc7892; Santa Cruz Biotechnology, Dallas, TX, USA), rabbit polyclonal anti-iNOS (1:100; ab3523; Abcam, Cambridge, UK) and anti-ET-1 (1:100; ab1568; Abcam, Cambridge, UK). Subsequently, the sections were incubated for one hour with secondary antibodies (anti-rabbit-HRP) for TGF-β, iNOS, and ET-1. Finally, immunostaining was developed with the DAB-Plus substrate Kit (00–2020, Life Technologies, Waltham, MA, USA) until brown staining was observed, and the sections were stained with Harris’ hematoxylin, dehydrated, and mounted with a synthetic resin. In the case of 8-OHdG, tissue sections were previously treated with proteinase K 10 µm/ml, followed by treatment with RNase 200 µg/ml. DNA was denatured with 2 N HCl, and the reaction was neutralized with 1 M Tris base. After blocking nonspecific sites with 5% BSA, the primary monoclonal mouse anti-8-OHdG antibody (1:200; GTX41980; GeneTex, Irvine, TX, USA) was added. Subsequently, the same protocol was followed as in the previous immunohistochemical analysis. Tissues were observed under an optical microscope at magnifications of 10x and 40x.

***Statistical analysis***

Specialized ImageJ software was used for quantification of the histological sections, and GraphPad Prism9 software (GraphPad, San Diego, CA, USA) was used for statistical analysis of the results. A pairwise comparison was performed using an unpaired Student’s t test. The results are shown as the means ± standard deviation (SD). A *P*<0.05 was considered statistically significant.

## Results

***Histological alterations in kidneys after BLM administration***

**Figure 1:**
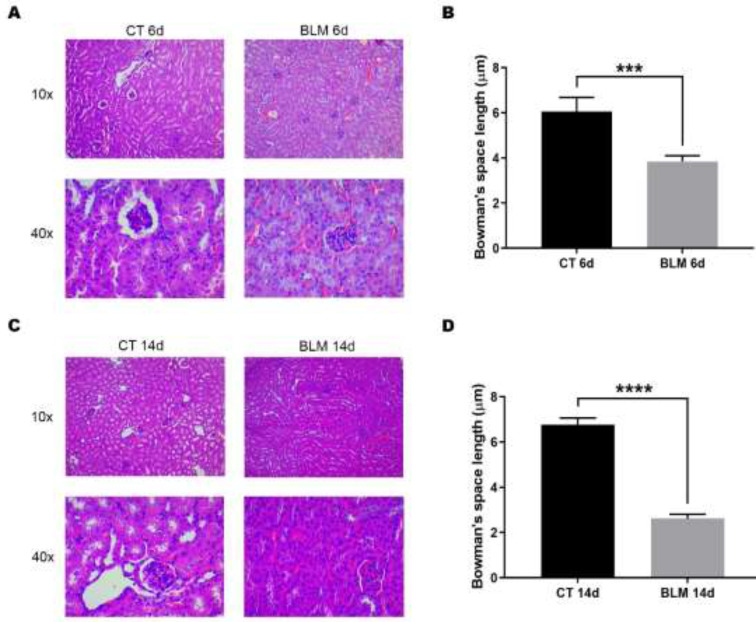
Histological staining of kidney tissue sections from control and BLM-treated mice

Before evaluating the effects of the BLM-induced SSC model in the kidneys, we performed validation of the induction of SSc by evaluating the presence of fibrosis in the skin and lungs. Histological analysis through Masson’s trichrome staining confirmed the induction of fibrosis in these organs, which was denoted by an increase in collagen deposition (Supplementary 1). Subsequently, to evaluate the possible histological alterations in the kidneys during the establishment of the experimental model of BLM-induced SSc, H&E staining was performed. The results showed that the kidneys of the BLM-treated group were histologically characterized by thickening of the renal corpuscles and different types of renal tubules. In addition, an alteration of the renal architecture indicated by the loss of the uniform distribution of the remaining renal tubules was observed (Figure 1A, C). Changes in renal histology, such as decreased Bowman’s space length compromise renal functionality (22). Our results showed that Bowman’s space length was significantly decreased to 3.8 μm (*P*<0.001) and 2.6 μm (*P* < 0.0001) at 6 and 14 days after BLM treatment compared with their control groups, whose length was 6.1 μm and 6.7 μm, respectively (Figure 1B, D). 

***BLM treatment increases the expression of the kidney damage marker ET-1***

**Figure 2:**
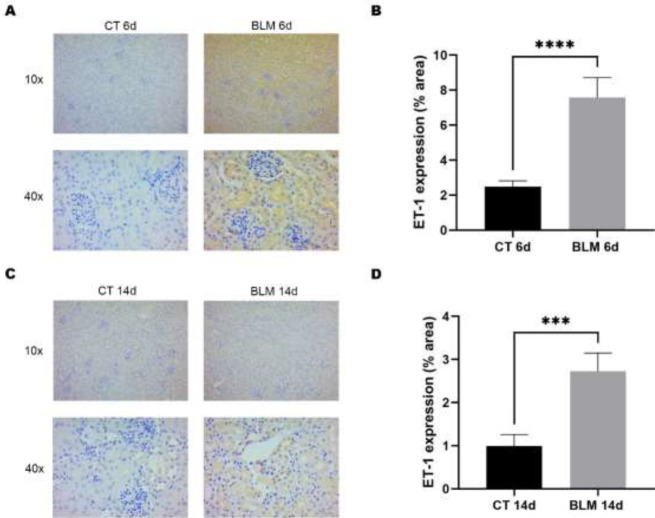
Immunohistochemical analysis of the renal damage marker ET-1

Due to its central role in the pathogenesis of kidney damage and the evaluation of its expression in biopsies from patients with SRC (23), ET-1 was evaluated as a marker of endothelial damage by immunohistochemistry. The results showed that the kidneys of BLM-exposed mice had increased ET-1 expression in the different renal tubules (Figure 2A, C); this increase was statistically significant at Days 6 (7.5%, *P*<0.0001) and 14 (2.7%, *P*<0.001) after BLM treatment compared to the control groups, with the Day 6 group showing the most significant increase (Figure 2B, D).

***BLM treatment increases oxidative stress levels through iNOS expression***

**Figure 3:**
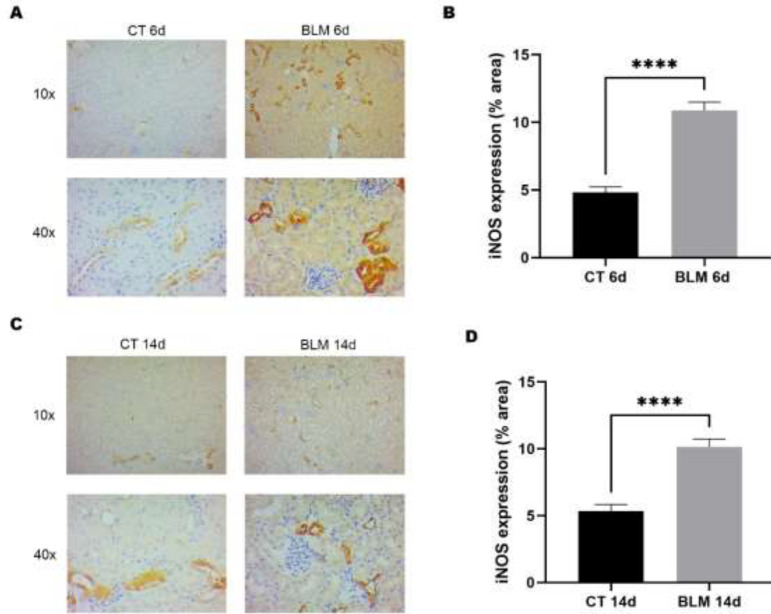
Immunohistochemical analysis of iNOS

An important feature of the BLM-induced SSc model is the generation of reactive oxygen species (ROS) and reactive nitrogen species (RNS). One of the enzymes responsible for NO production is iNOS; therefore, the expression of this enzyme was evaluated by immunohistochemistry. The results indicated that BLM-treated groups showed an increase in iNOS expression, which was mainly observed in renal tubules (Figure 3A, C) and was statistically significant in BLM-treated groups at 6 (10.8%, *P*<0.0001) and 14 (10.1%, *P*<0.0001) days compared to their respective control groups (Figure 3B, D).

***BLM treatment increases oxidative DNA damage in the kidney***

**Figure 4:**
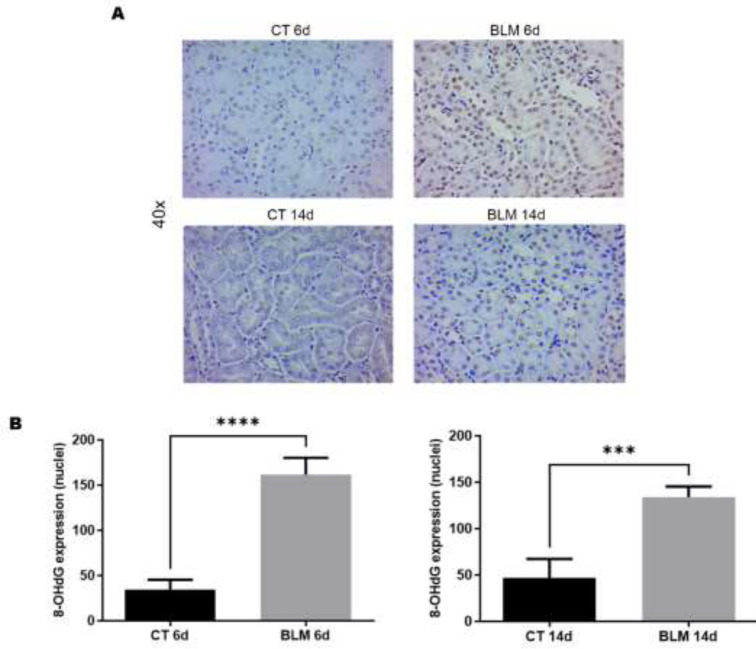
Immunohistochemical analysis of the DNA damage marker 8-OHdG

Once we determined the presence of oxidative stress in the kidneys after BLM administration and because it is known that ROS can oxidize and damage DNA, we proceeded to evaluate 8-OHdG as a marker of oxidative DNA damage. The results showed an increase in 8-OHdG expression in the BLM-treated groups (Figure 4A). Positive nuclei were subsequently quantified, and a statistically significant increase was observed in the BLM-treated groups at 6 (161 nuclei, *P*<0.0001) and 14 (133 nuclei, *P*<0.001) days compared to controls (Figure 4B, C).

***BLM treatment induces expression of the profibrotic protein TGF-*** ***β***

**Figure 5:**
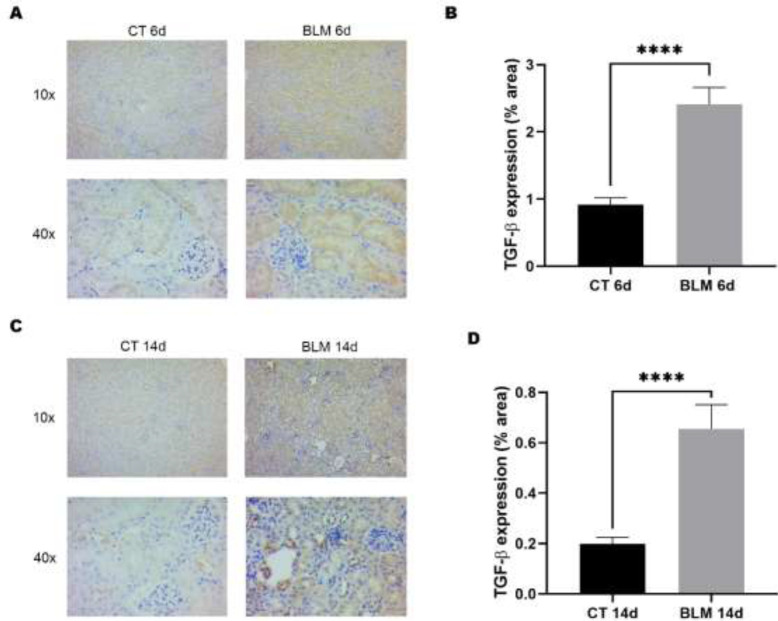
Immunohistochemical analysis of the profibrotic marker TGF-β

TGF-β expression is consistently elevated in affected organs in SSc and correlates with increased extracellular matrix deposition; in this regard, we proceeded to evaluate TGF-β expression in kidneys by immunohistochemistry. The results showed an increase in the expression of this protein in the groups treated with BLM (Figure 5A, C). After immunohistochemistry, we proceeded to quantify the TGF-β-positive area. The results showed a statistically significant increase in the groups treated with BLM sacrificed at 6 (2.4%, *P*<0.0001) and 14 (0.6%, *P*<0.0001) days compared to their respective control groups (Figure 5B, D).

***BLM treatment promotes extracellular matrix deposition in the kidney***

**Figure 6:**
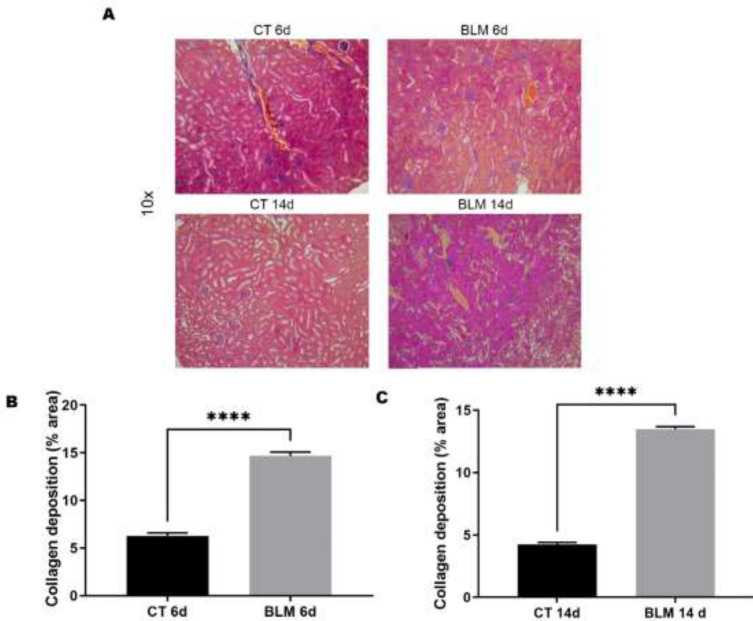
Use of Masson's trichrome staining to assess collagen deposition

Fibrosis is characterized by excessive ECM deposition and is a common pathological feature of SSc. Therefore, it is extremely important to assess collagen deposition in the kidneys during SSc because collagen is one of the main components of the ECM. Therefore, we assessed collagen deposition by Masson’s trichrome staining. The results showed that BLM-treated groups had higher collagen deposition mainly in renal tubules, glomeruli, and around Bowman’s capsule (Figure 6A). Quantification of the collagen-positive area was performed as previously described (21). The results showed that this increase in collagen deposition was statistically significant in the BLM-treated groups sacrificed at 6 (14.6%, *P*<0.0001) and 14 (13.4%, *P*<0.0001) days compared to their respective control groups (Figure 6B, C).

(A, C) Representative images of H&E staining of CD1 mouse kidney sections at Days 6 and 14 after saline (CT) or BLM administration. Top (10x), bottom (40x). (B, D) Quantification of Bowman's space length at 6 and 14 days after treatment. The *P-*value is shown as ***P<0.001; ****P<0.0001. The graphs show the means ± SD for all groups, n=4

BLM: bleomycin

(A, C) Representative images of ET-1 immunohistochemistry in mouse kidney sections at Days 6 and 14 after saline (CT) or BLM administration. (B, D) Quantification of ET-1 expression levels at 6 and 14 days after treatment. The *P-*value is shown as ***P<0.001; ****P<0.0001. Graphs show the means ± SD of all groups, n=4

ET-1: endothelin-1; BLM: bleomycin

(A, C) Representative images of immunohistochemistry for iNOS in mouse kidney sections at Days 6 and 14 after saline (CT) or BLM administration. (B, D) Quantification of iNOS expression levels at 6 and 14 days after treatment. The *P*-value is shown as ***P <0.001; ****P<0.0001. Graphs show the means ± SD of all groups, n=4

iNOS: inducible nitric oxide synthase; BLM: bleomycin

(A) Representative images of immunohistochemistry for 8-OHdG in mouse kidney sections at Days 6 and 14 after saline (CT) or BLM administration. (B, C) Quantification of 8-OHdG-positive nuclei at 6 and 14 days after treatment. The *P*-value is shown as ***P<0.001; ****P<0.0001. Graphs show the means ± SD of all groups, n=4

8-OHdG: 8-hydroxy-2-deoxyguanosine; BLM: bleomycin

(A, C) Representative images of immunohistochemistry for TGF-β in mouse kidney sections at Days 6 and 14 after saline (CT) or BLM administration. (B, D) Quantification of TGF-β expression levels at 6 and 14 days after treatment. The *P*-value is shown as ***P<0.001; ****P<0.0001. Graphs show the means ± SD of all groups, n=4

TGF-β: transforming growth factor β; BLM: bleomycin

(A) Representative images of Masson's trichrome staining of mouse kidney sections at Days 6 and 14 after saline (CT) or BLM administration. (B, C) Quantification of collagen deposition at 6 and 14 days after treatment. The *P*-value is shown as ***P<0.001; ****P<0.0001. Graphs show the means ± SD of all groups, n=4

BLM: bleomycin

## Discussion

Although considerable progress has been made in understanding the pathophysiology of SSc, the molecular mechanisms by which renal damage develops remain unclear. Murine models of SSc are a crucial tool to investigate pathophysiological mechanisms (24). Therefore, the present study evaluated alterations in the kidneys in a model of SSc induced by BLM administered by osmotic minipump implantation. This model has successfully replicated the development of fibrosis in organs such as skin and lungs and has also contributed to the identification of the central cytokines, growth factors, and signaling pathways involved in the development of SSc, as well as the identification of the main histological changes that occur in SSc (14, 19, 25). However, no studies have assessed the damage that occurs in the kidneys during establishment of this model; therefore, this study provides evidence about renal injury in the BLM-induced SSc model, which could be helpful for future research.

One of the main effects of BLM damage is observed on endothelial and epithelial cells, producing morphological alterations (15). Histology revealed a prominent juxtaglomerular apparatus known as arterial thrombosis, which is damage in the renal corpuscles denoted by inflammation of the glomeruli. This feature is strongly associated with glomerulonephritis that prevents proper filtration of blood, urine production and a decrease in the light that passes through the other renal tubules. These findings are consistent with the changes observed in the biopsies of patients with SRC (26, 27). However, in our staining, we could not observe onion-shaped lesions characteristic of SRC and thrombosis in the glomerular capillaries because other histological techniques are needed.

One of the mechanisms of damage caused by BLM is its ability to mediate DNA strand cleavage in the presence of iron and oxygen, producing single- or double-strand breaks with the consequent overproduction of ROS and RNS (28). Likewise, ROS are considered key to the pathology of disease and contribute significantly to the clinical manifestations associated with SSc (12). Studies in animal models have further strengthened the hypothesis of a role for oxidative stress in the onset and course of this disease. These reports correlate with the results obtained in our investigation. We observed an increase in iNOS expression in the BLM-treated groups, which may suggest an increase in nitric oxide in the kidneys. On the other hand, we evaluated the presence of oxidative damage to DNA through the expression of 8-OHdG, and our results report an increase in 8-OHdG in kidneys after BLM administration; these findings correlate with those found by Fujita and collaborators who reported a rise in 8-OHdG in lungs after BLM administration (29). This suggests that BLM-treated mice have an altered redox state.

It has been suggested that damage to the microvasculature caused by increased vasoconstriction is a key feature of the pathophysiological model of SRC. ET-1 is an endogenous vasoconstrictor produced by vascular endothelial cells, mediating vascular proliferation, fibrosis, and inflammation (30). In 2011, a study reported that ET-1 is overexpressed in microangiopathic lesions in glomeruli, arterioles, and interlobular arteries in renal biopsy specimens obtained from 14 patients with SRC (23), consistent with the results obtained in our study. However, a more significant increase in ET-1 was observed in mice treated with BLM sacrificed at 6 days compared to mice sacrificed at 14 days; this may be because one of the mechanisms to control ET-1 is NO release. Furthermore, NO is considered to have a biphasic effect in physiological and pathological conditions, being beneficial and detrimental depending on the concentration and local environment (31).

The fibroblast-to-myofibroblast transition is crucial for the development of SSc because myofibroblasts induce the fibrotic process due to their high capacity for ECM synthesis (32). In this regard, several cytokines stimulate this transition, including TGF-β, whose role is relevant for the development of fibrosis (33). Several studies have reported a central role for TGF-β in the pathophysiology of the disease, proposing it as a therapeutic target or as a biomarker for SSc (34-36). In this work, we demonstrated that TGF-β is overexpressed in the renal tissue of BLM-treated mice, suggesting the involvement of TGF-β in the pathophysiology of SSc-associated renal damage.

The combination of different factors, such as endothelial cell damage, inflammation, oxidative stress and increased profibrotic factors, triggers ECM deposition, mainly collagen; therefore, collagen deposition was evaluated in our model by Masson’s trichrome staining. In addition, several authors have pointed to myofibroblasts as the main ECM producers (32, 37, 38). In this sense, it is worth mentioning that renal myofibroblasts are mainly located in the renal interstitium (the intertubular area between nephrons, urethral epithelium and renal vasculature) under fibrotic conditions and originate from local fibroblasts, pericytes or fibrocytes derived from infiltrating bone marrow (39). This background is consistent with the sites where collagen deposition was concentrated in the renal tissue sections of our model. Therefore, these results suggest that, in the BLM-induced SSc model, there is an increase in collagen in addition to changes in kidney histology.

## Conclusion

We demonstrated that the BLM-induced SSc model administered using an osmotic minipump produces histological alterations in the kidneys, increased collagen deposition, and overexpression of proteins involved in microvasculopathy, oxidative stress, inflammation, and fibrosis. Furthermore, our results show that the BLM-induced SSc model could be helpful to study the molecular mechanisms involved in the development of renal damage in patients with SSc.

## Authors’ Contributions

VRVG, SVT, and RBH study conception and designed the experiments; DCPF, ERJ and I RA, perform experiment; DCPF, ERJ, IRA, JMVE, HTA, and KGG analysis and interpretation of results; DCPF, ERJ, IRA, JMVE, and KGG draft manuscript preparation; VRVG, SVT, HTA, and RBH critical revision y editing of the article; VRVG, RBH, DCPF, ERJ, IRA, JMVE, SVT, HTA, and KGG final approval of the version to be published.

## Conflicts of Interest

The authors declare that they have no conflicts of interest.
